# Use of Porphyrin Precursors as a Novel Approach for Detection of *Mycobacterium tuberculosis*

**DOI:** 10.3390/ijms27188227

**Published:** 2026-09-15

**Authors:** Oganes A. Ambartsumyan, Platon I. Eliseev, Olesya A. Skuredina, Ekaterina Y. Gosteva, Tatiana E. Tiulkova, Anna E. Panova, Anastasia G. Samoilova, Irina A. Vasilyeva

**Affiliations:** 1 National Medical Research Center of Phthisiopulmonology and Infectious Diseases, Ministry of Health of the Russian Federation, Moscow 127473, Russia; 2 Osipyan Institute of Solid State Physics RAS, Chernogolovka 142432, Russia

**Keywords:** tuberculosis, tuberculosis diagnostics, *Mycobacterium tuberculosis*, pathogen detection, biomarkers, porphyrins, metabolites, fluorescence spectroscopy

## Abstract

Tuberculosis remains one of the leading infectious diseases worldwide, which is why new approaches are being developed as a foundation for future diagnostic methods. One promising strategy relies on porphyrins as biomarkers for the detection of *Mycobacterium tuberculosis* using fluorescence spectroscopy. Previous attempts have been made to detect microorganisms based on their porphyrin production. However, insufficient endogenous porphyrin production limits the applicability of this approach for mycobacterial detection. As a proof-of-principle study, we investigated the feasibility of using exogenous precursors to enhance porphyrin accumulation with the aim of detecting mycobacterial growth by means of fluorescence spectroscopy. The introduction of these precursors resulted in a significantly higher fluorescence signal intensity, making it possible to detect *Mycobacterium tuberculosis* and identify potential contamination.

## 1. Introduction

Tuberculosis (TB) is one of the leading infectious diseases worldwide. It is caused by *Mycobacterium tuberculosis* (*Mtb*), which is estimated to infect approximately one quarter of the global population. According to the WHO, in 2024, 10.7 million people developed TB; the global average incidence was 131 cases per 100,000 population, and 1.23 million deaths were attributed to the disease. TB has thus once again become the leading cause of death from a single infectious agent, surpassing COVID-19 [1]. Currently, various methods are used to detect *Mtb*, including molecular, culture-based, and microscopic techniques. Together, these approaches form a diagnostic framework that enables detection of most tuberculosis cases.

Sputum smear microscopy remains an important tool for TB diagnosis because it provides rapid results, is simple to perform, and has high specificity [2]. However, its key limitations include low sensitivity, the inability to distinguish *Mtb* from other acid-fast microorganisms, such as nontuberculous mycobacteria (NTM), and the lack of information on the drug susceptibility of the causative agent [3,4].

Molecular methods, particularly PCR-based assays, enable rapid detection of *Mtb* directly in clinical specimens with high sensitivity and specificity and can also identify mutations associated with drug resistance [5,6,7].

Culture-based methods using solid and liquid media are among the standard diagnostic approaches for TB. They allow for the isolation of a pure *Mtb* culture and subsequent drug susceptibility testing [8]. However, these methods are characterized by a long time to result and a limited ability to differentiate *Mtb* from NTM [9].

Automated liquid culture systems, such as the BACTEC MGIT 960 system, which detects mycobacterial growth by measuring the fluorescence of an oxygen-sensitive sensor embedded in the tube (a ruthenium metal complex whose fluorescence is quenched by dissolved oxygen and increases as oxygen is consumed by growing microorganisms), reduce time to detection by measuring fluorescence proportional to mycobacterial growth. However, they require costly equipment and reagents and do not eliminate the need for prolonged incubation or the inability to reliably distinguish *Mtb* from other bacterial species. These limitations highlight the need to develop new, faster, and highly specific diagnostic methods [10,11].

The life cycle of mycobacteria involves the metabolism of a wide range of biological compounds, which may serve as targets for detection and be considered biomarkers of the causative agent of TB [12]. In this context, new diagnostic approaches based on the analysis of metabolites and biomarkers are being actively explored to improve the sensitivity, specificity, and speed of *Mtb* detection [13].

Among various bacterial biomarkers, porphyrins are of particular interest. These tetrapyrrolic macrocyclic compounds play a key role in the metabolism of many microorganisms. Porphyrins are precursors of heme, a component of the respiratory chain that supports cellular redox processes [14].

**Figure 1:**
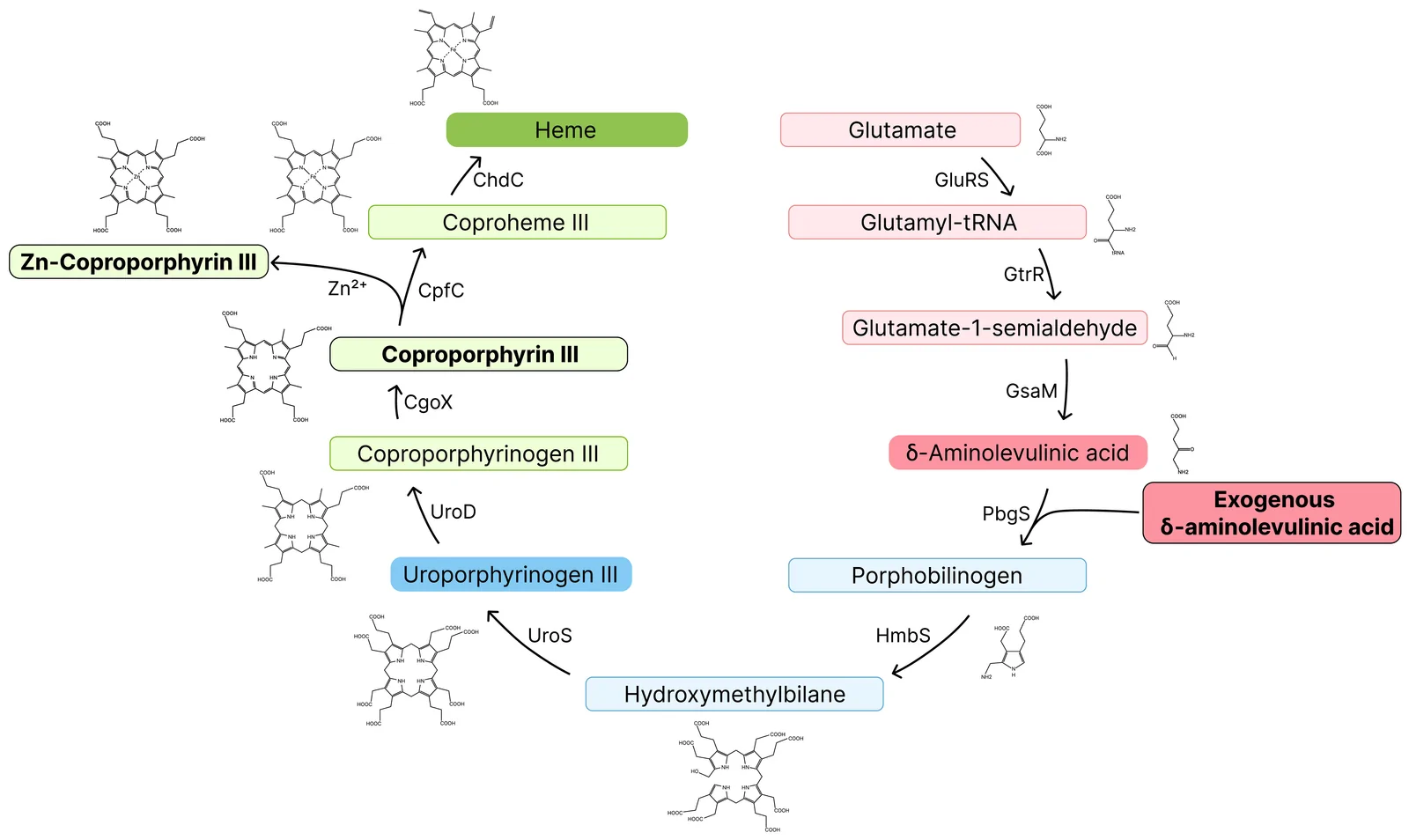
The *M. tuberculosis* heme biosynthesis pathway.

In most bacteria, heme biosynthesis is initiated via the C5 pathway, in which heme precursors are derived from glutamate (Figure 1). At the terminal stages of porphyrin ring synthesis, porphyrins can be produced through two principal routes, depending on taxonomic group: a protoporphyrin-dependent pathway, characteristic of most Gram-negative bacteria and eukaryotes, and a coproporphyrin-dependent pathway, typical of Gram-positive bacteria, including mycobacteria [14,15,16,17].

Key steps of the CPD pathway include (1) synthesis of 5-aminolevulinic acid and formation of uroporphyrinogen III; (2) decarboxylation of uroporphyrinogen III to coproporphyrinogen III; (3) oxidation of coproporphyrinogen III to coproporphyrin III; (4) metallation of coproporphyrin III to form coproheme III; and (5) oxidative decarboxylation of coproheme III to heme.

The presence of porphyrins in mycobacteria was demonstrated as early as the mid-twentieth century in the causative agents of human and avian TB, providing the biochemical rationale for the subsequent development of fluorescence-based methods for their detection [18,19].

Endogenous porphyrins produced by microorganisms, including mycobacteria, exhibit intense fluorescence in the red spectral region upon excitation within the Soret band (~400–410 nm), where porphyrins display their strongest absorption [16,20,21,22]. The fluorescence spectrum exhibited three characteristic emission maxima at 580, 620, and 685 nm. Among these, the 620 nm peak showed the highest fluorescence intensity under the experimental conditions used. The 580 nm peak was attributed to Zn-coproporphyrin, whereas the peaks at 620 and 685 nm were attributed to free porphyrins, predominantly coproporphyrin III in *Mtb* [23,24,25,26,27,28,29,30].

However, it should be noted that endogenous porphyrins are synthesized slowly, which is an important limiting factor for their use in mycobacterial detection. To accelerate porphyrin synthesis, exogenous inducers that affect endogenous porphyrin production in mycobacteria may be used [31,32].

Based on the established ability of 5-aminolevulinic acid hydrochloride (ALA) to induce porphyrin synthesis in bacterial cells, we hypothesized that mycobacteria and fast-growing contaminating microorganisms differ in the kinetics and spectral profile of ALA-induced porphyrin accumulation, and that these differences can be exploited to register mycobacterial growth and to detect culture contamination. Accordingly, this study evaluated, as a proof of principle, the feasibility of using exogenous porphyrin-biosynthesis precursors for this purpose by monitoring the 620 nm fluorescence intensity and the 580/620 nm ratio in *Mtb* H37Rv and in a panel of common contaminants incubated under identical conditions over 15 days.

## 2. Results

**Figure 2:**
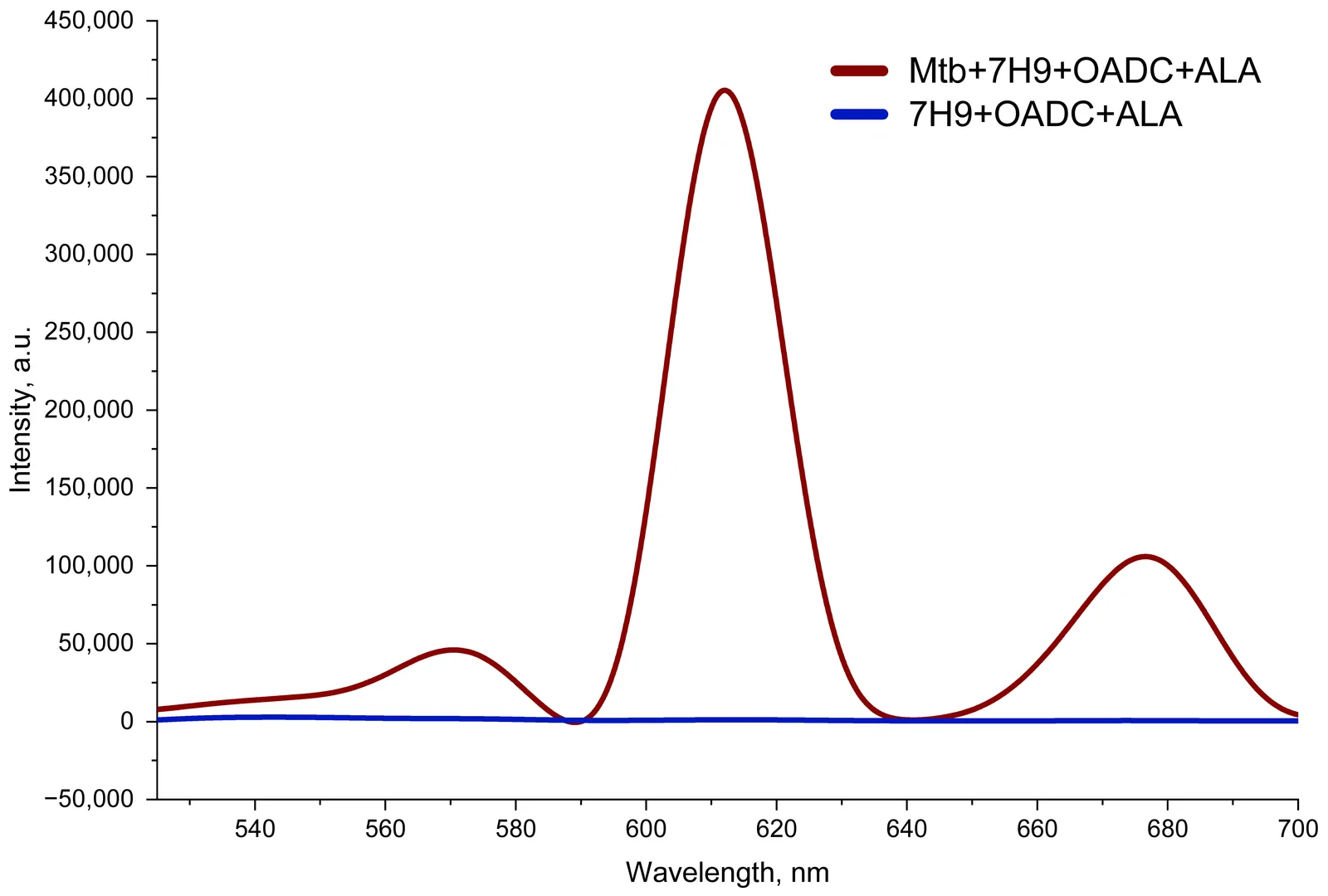
Fluorescence emission spectra of *Mycobacterium tuberculosis*. Fluorescence spectra were recorded daily at an excitation wavelength of 405 nm. The red line represents the bacterial culture supplemented with ALA, while the blue line represents the control without bacterial culture. Three characteristic emission maxima were observed at approximately 580, 620, and 685 nm, with the 620 nm peak showing the highest fluorescence intensity. The peak at 580 nm was assigned to Zn-coproporphyrin, while the peak at 620 nm was mainly associated with coproporphyrin III. The spectra demonstrate the progressive increase in porphyrin-associated fluorescence during *Mtb* cultivation. *Mtb*, *Mycobacterium tuberculosis*; nm, nanometers.

During fluorescence measurements and subsequent data analysis of *Mtb* samples, three characteristic fluorescence peaks were detected, with maxima at 580, 620, and 685 nm, as mentioned earlier. These spectral peaks were not observed in negative control samples containing oleic acid, albumin, dextrose, and catalase (OADC) enrichment and no *Mtb* (Figure 2). The fluorescence signal at 620 nm showed the highest intensity and a linear relationship with culture time, which provided the basis for considering this peak as the primary marker for monitoring mycobacterial growth.

**Figure 3:**
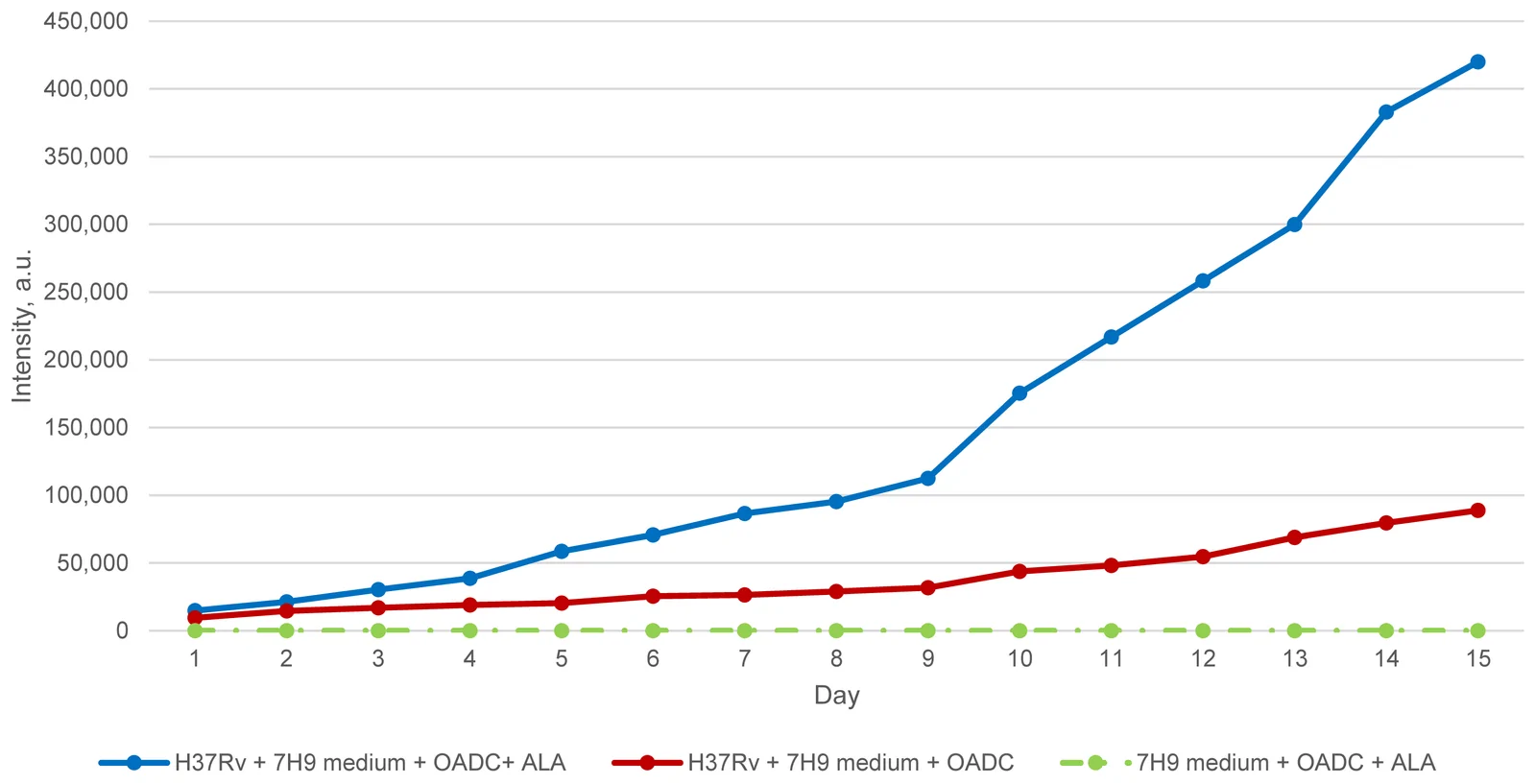
Effect of the porphyrin precursor on fluorescence signal intensity. Fluorescence intensity was measured daily at 620 nm in cultures supplemented with ALA and in cultures without ALA. The blue line represents the culture supplemented with ALA, the red line represents the culture without ALA, and the green line represents the control containing ALA without bacterial culture. ALA supplementation resulted in a significantly greater increase in fluorescence intensity compared with cultures without ALA. Ten independent samples (*n* = 10) were analyzed for each microorganism at each time point. Data are presented as median fluorescence intensity values. ALA, 5-aminolevulinic acid; *Mtb*, *Mycobacterium tuberculosis*; nm, nanometers.

In experiments evaluating the effect of ALA on fluorescence at 620 nm during the cultivation of *Mtb*, the presence of ALA significantly accelerated the increase in fluorescence intensity and resulted in a higher overall signal compared to samples without ALA (*p* < 0.01) (Figure 3). No fluorescence signal was detected in negative control samples.

**Figure 4:**
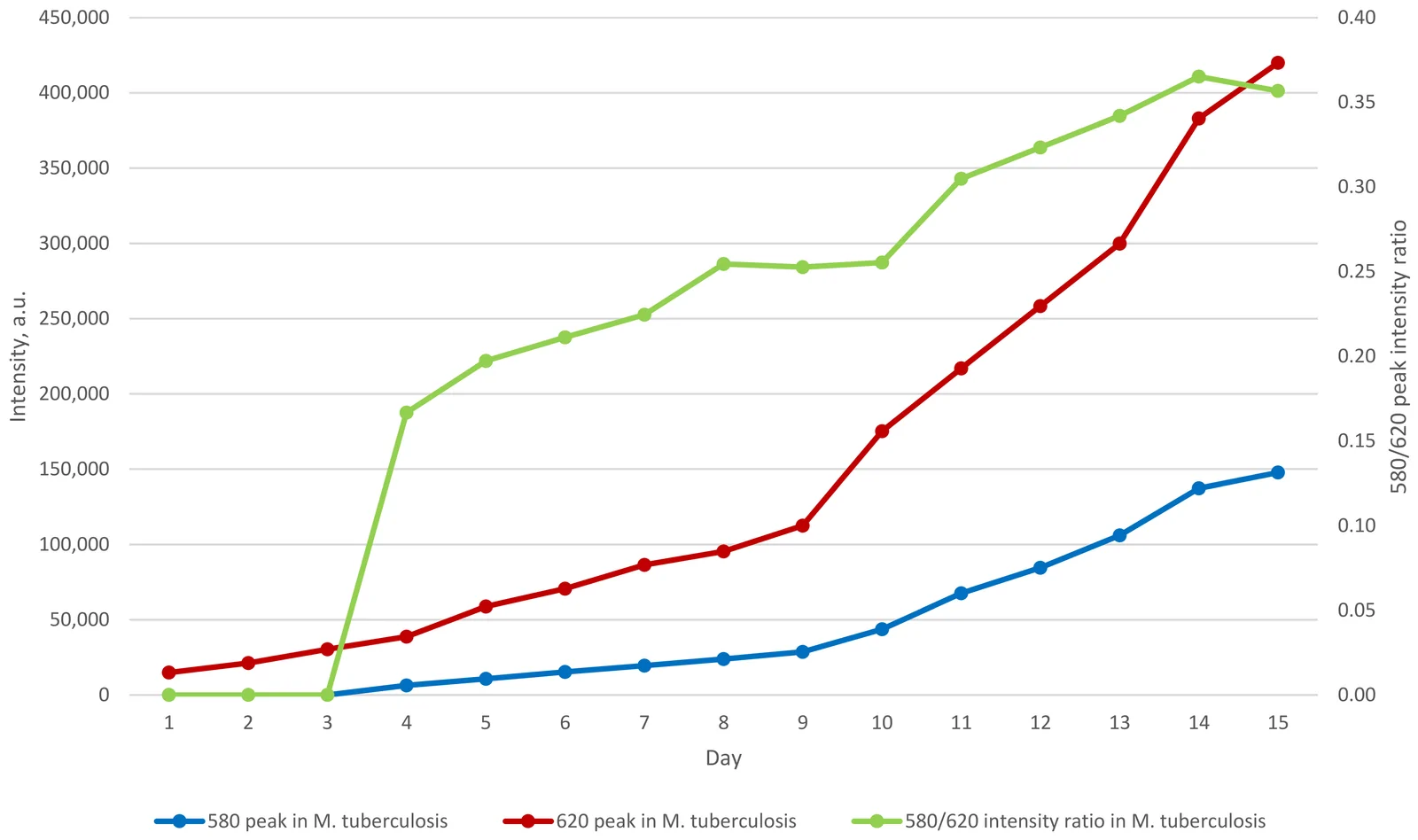
Median fluorescence signal values during *Mtb* culture. Fluorescence signals were recorded daily at 580 (blue line) and 620 nm (red line) for 15 days of cultivation. The 620 nm fluorescence signal predominated throughout the observation period and increased progressively during cultivation. The 580/620 nm fluorescence intensity ratio (green line) increased from 0 on day 1 to 0.36 on day 15, reflecting the changing contribution of the two fluorescence peaks. Ten independent samples (*n* = 10) were analyzed for each microorganism at each time point. Data are presented as median values. *Mtb*, *Mycobacterium tuberculosis*; nm, nanometers.

To detect growth of *Mtb*, fluorescence was measured daily, with signal intensities recorded at 580 and 620 nm, along with the peak ratio. During the first 7 days of culture, the median value of this ratio did not exceed 0.22, whereas by day 15 it increased to 0.36. These findings indicate the predominant contribution of the 620 nm peak (Table 1, Figure 4).

**Figure 5:**
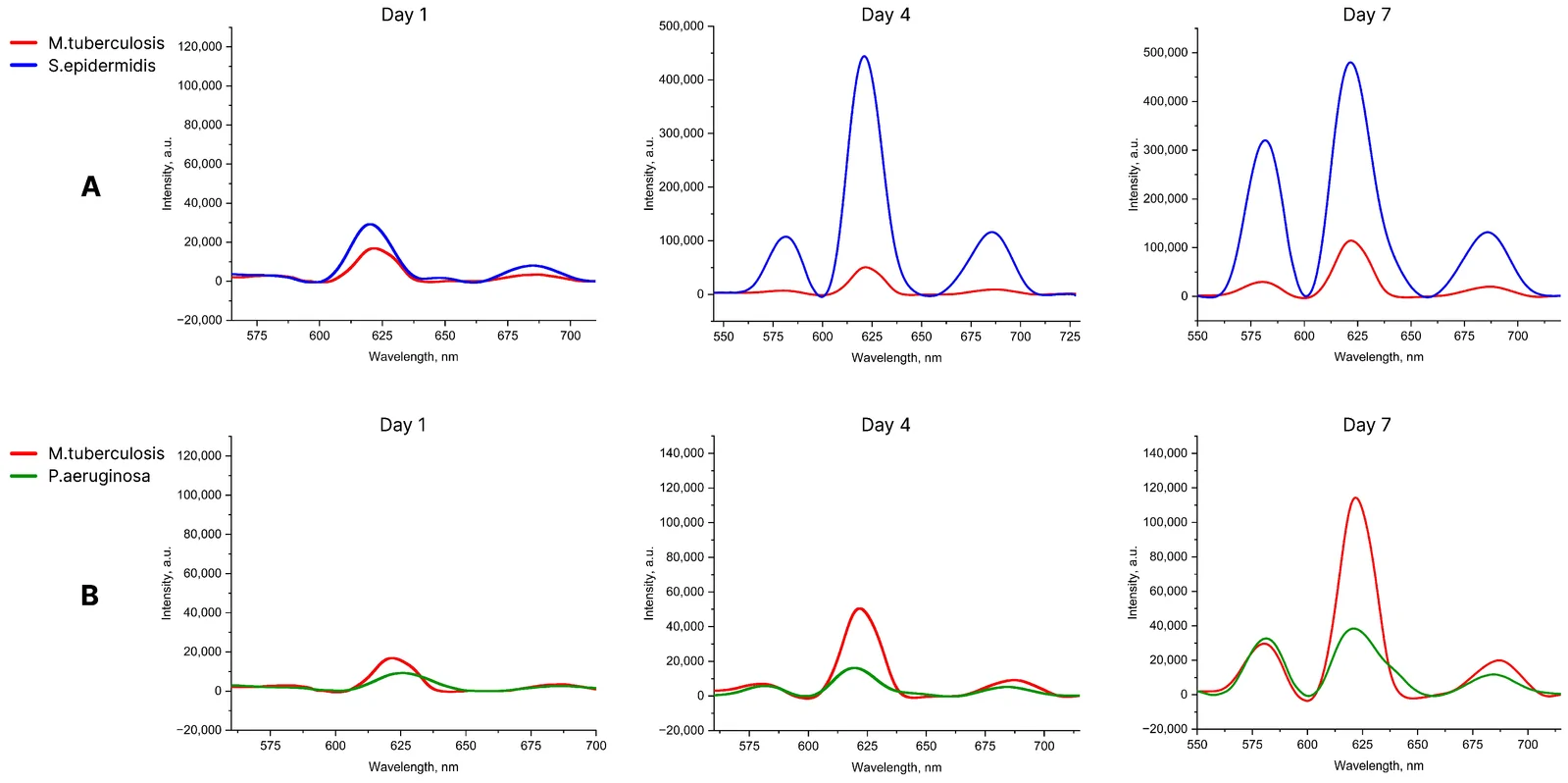
Differences in the spectral characteristics of *Mtb* and other bacteria over time: (**A**) Fluorescence intensity at 620 nm and (**B**) the 580/620 nm fluorescence peak intensity ratio measured on days 1, 4, and 7 of cultivation. The red line represents *Mycobacterium tuberculosis* H37Rv, the blue line represents *Staphylococcus epidermidis*, and the green line represents *Pseudomonas aeruginosa*. The fluorescence characteristics differed between *Mtb* and the tested non-mycobacterial microorganisms, with the 620 nm fluorescence intensity and the 580/620 nm ratio providing complementary parameters for their differentiation. Data are presented as median values; *n* = 10 independent experiments per microorganism. Statistical significance was assessed using a linear mixed-effects model, with *p* < 0.05 considered statistically significant. *Mtb*, *Mycobacterium tuberculosis*; nm, nanometers.

To further evaluate the potential of this approach for differentiating *Mtb* from other opportunistic microorganisms, an additional experiment was performed using cultures of *S. aureus*, *S. epidermidis*, *P. aeruginosa*, *K. pneumoniae*, and *C. albicans*. Differentiation relied on two complementary parameters: the growth dynamics of the 620 nm peak (Figure 5A) and the 580/620 nm peak intensity ratio (Figure 5B).

**Figure 6:**
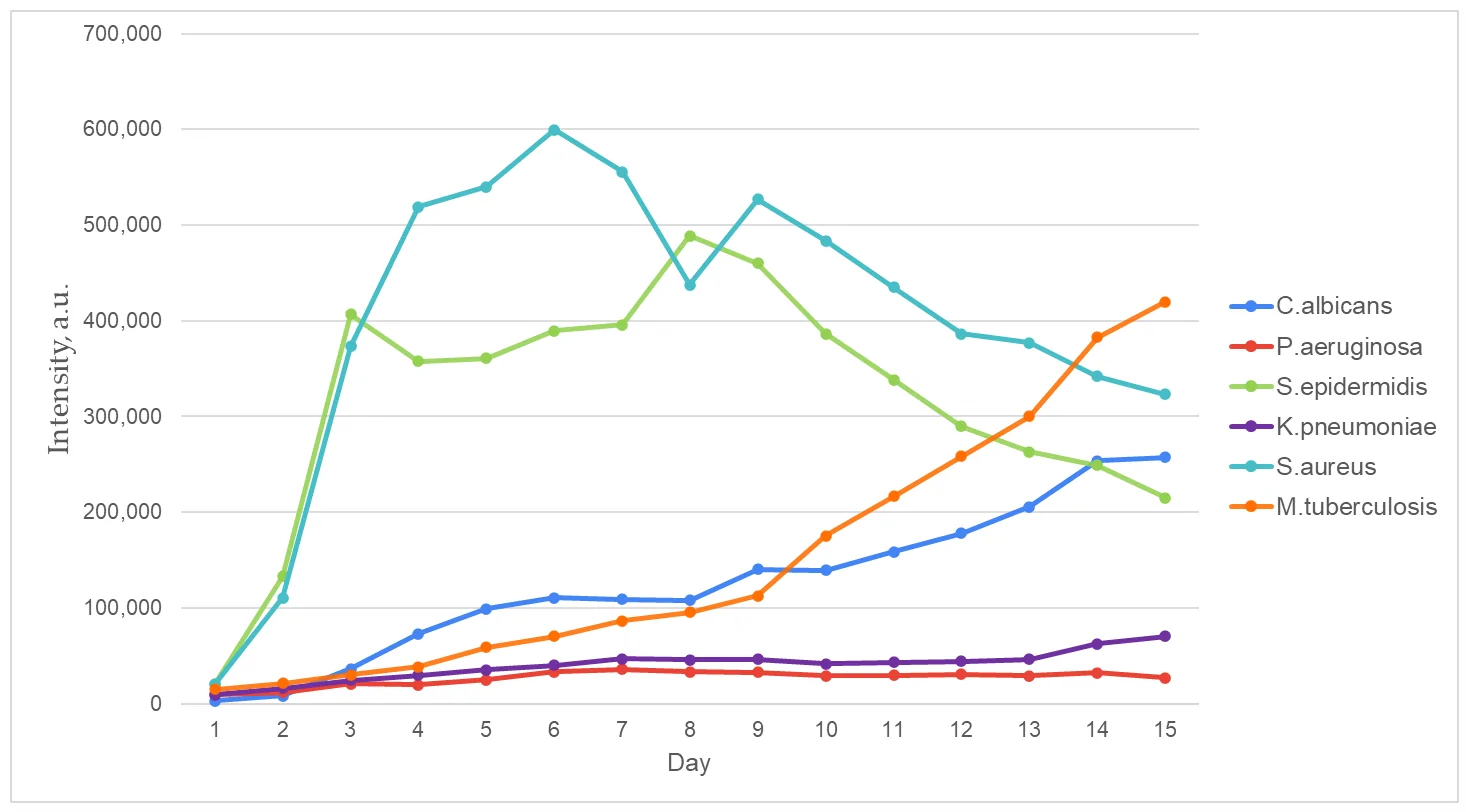
Median fluorescence signal intensities at 620 nm by day. Fluorescence intensity was measured daily for 15 days (horizontal axis) in *Mycobacterium tuberculosis* H37Rv (orange line), *Staphylococcus epidermidis* (green line), *Staphylococcus aureus* (cyan line), *Klebsiella pneumoniae* (purple line), *Pseudomonas aeruginosa* (red line), and *Candida albicans* (blue line). Ten independent samples (*n* = 10) were analyzed for each microorganism at each time point. The dynamics of the 620 nm fluorescence signal differed between *Mtb* and the tested microorganisms, with particularly pronounced early increases observed for *S. epidermidis* and *S. aureus*. Data are presented as median fluorescence intensity values. Statistical significance was assessed using two-way repeated-measures analysis of variance (ANOVA) with species and time as factors; *p* < 0.05 was considered statistically significant. *Mtb*, *Mycobacterium tuberculosis*; nm, nanometers.

**Figure 7:**
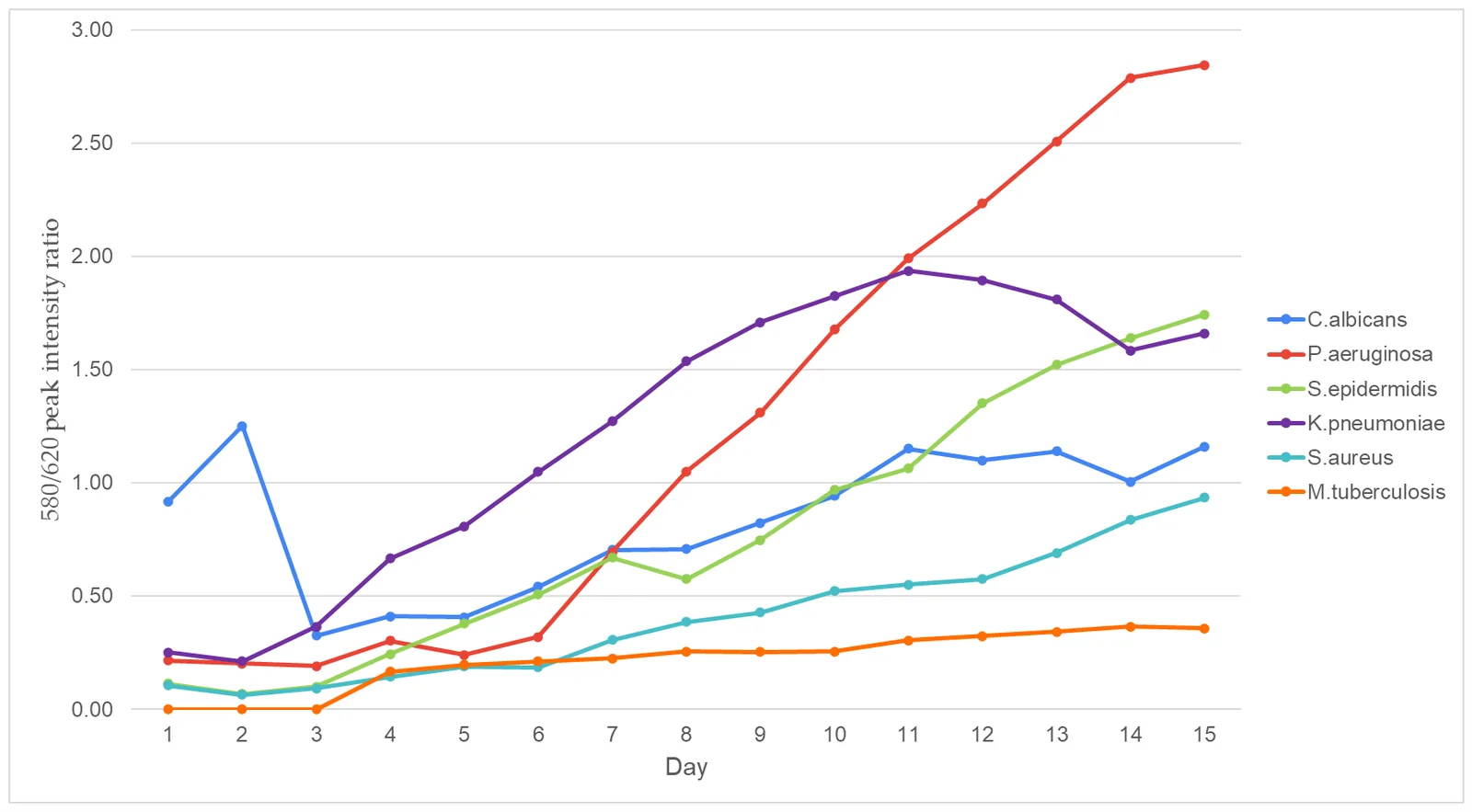
Median 580/620 nm peak intensity ratio by day. Fluorescence intensity was measured daily for 15 days (horizontal axis) in *Mycobacterium tuberculosis* H37Rv (orange line), *Staphylococcus epidermidis* (green line), *Staphylococcus aureus* (cyan line), *Klebsiella pneumoniae* (purple line), *Pseudomonas aeruginosa* (red line), and *Candida albicans* (blue line). During the 15-day observation period, the ratio remained below 0.35 in *Mycobacterium tuberculosis* H37Rv, whereas it increased to approximately 1.0 or higher in the tested opportunistic microorganisms. This parameter therefore provided a differential fluorescence characteristic for *Mtb* compared with the tested contaminants. Ten independent samples (*n* = 10) were analyzed for each microorganism at each time point. Data are presented as median values. Statistical significance was assessed using a linear mixed-effects model; *p* < 0.05 was considered statistically significant. *Mtb*, *Mycobacterium tuberculosis*; nm, nanometers.

The intensity of the 620 nm peak in *S. epidermidis* began to increase on day 2 of observation and reached its maximum on day 4 and overall was higher than in *Mtb* (*p* < 0.01) (Table 2; Figure 6). The 580/620 peak intensity ratio was also significantly higher in *S. epidermidis* (*p* < 0.01), indicating a predominant increase in the 580 nm peak relative to the 620 nm peak during dynamic monitoring of *S. epidermidis* culture (Figure 7). On day 15, the signal ratio in *S. epidermidis* exceeded 3, whereas in *Mtb* it was 0.34.

The intensity of the 620 nm peak in *S. aureus* began to increase on day 2 and reached its maximum on day 4, as was observed for *S. epidermidis*. On day 4, the 620 nm signal intensity in *S. aureus* was 18-fold higher than the corresponding value in *Mtb* (*p* < 0.01) (Table 3; Figure 6). The 580/620 peak intensity in *S. aureus* exceeded 0.9 on day 15, compared with 0.34 in *Mtb* (*p <* 0.05) (Figure 7).

The intensity of the 620 nm peak in *K. pneumoniae* showed dynamics comparable to those observed for *Mtb*, increasing from day 1 and reaching a maximum by day 15 (Table 4; Figure 6), and overall was lower than in *Mtb* (*p* < 0.01). However, the 580/620 peak intensity ratio was significantly higher in *K. pneumoniae* (*p* < 0.01), indicating a predominant increase in the 580 nm peak relative to the 620 nm peak during dynamic monitoring of *K. pneumoniae* culture. On day 15, the signal ratio in *K. pneumoniae* was 2.4, whereas in *Mtb* it was 0.34 (Figure 7).

The intensity of the 620 nm peak in *C. albicans* showed dynamics comparable (*p* > 0.05) to those observed for *Mtb*, increasing from day 1 and reaching a maximum by day 15 (Table 5; Figure 6). The 580/620 peak intensity ratio was significantly higher in *C. albicans* (*p* < 0.001), indicating a predominant increase in the 580 nm peak relative to the 620 nm peak during dynamic monitoring of *C. albicans* culture. On day 15, the signal ratio in *C. albicans* was 1.5, whereas in *Mtb* it was 0.34 (Figure 7).

Although the 620 nm peak signal was significantly lower for *P. aeruginosa* than in *Mtb (p* < 0.01), the 580/620 peak intensity ratio was significantly higher in *P. aeruginosa* than in *Mtb* (*p* < 0.01), indicating a predominant increase in the 580 nm peak relative to the 620 nm peak during dynamic culture monitoring (Table 6; Figure 6). On day 15, the signal ratio in *P. aeruginosa* was 4.4, whereas in *Mtb*, it was 0.34 (Figure 7).

## 3. Discussion

### 3.1. Porphyrin Fluorescence in Diagnostics and Photodynamic Applications

The selective use of ALA-induced porphyrin fluorescence described here is consistent with, and builds upon, a substantial body of previous work on bacterial porphyrins for diagnostic and therapeutic purposes in a variety of clinical contexts.

The work of Kilian represented the first description of the porphyrin test as a standalone diagnostic method for the genus *Haemophilus*. The approach, validated on 134 strains, was based on the ability of bacteria to synthesize porphyrins from exogenous ALA, thereby enabling indirect assessment of enzymatic activity along the heme biosynthetic pathway and, consequently, determination of X-factor requirement. The method eliminated a key limitation of the conventional satellite test—namely, its dependence on medium composition and carry-over of the X factor with the inoculum. The proposed detection scheme—recording of red fluorescence under UV illumination or colorimetric development with Kovacs reagent—was notable for its simplicity and reproducibility, facilitating its rapid adoption in clinical microbiology laboratories. This study provided the methodological foundation for the subsequent large-scale clinical validation of the method by Lund and Blazevic [33].

Lund and Blazevic evaluated the porphyrin test, based on the addition of ALA to the culture medium, as a rapid approach for differentiating clinical isolates of *Haemophilus* according to their X-factor requirement, comparing it with the conventional satellitism growth test. Their study demonstrated that detection of ALA-induced fluorescent porphyrins within 4 h provided a result that was both more rapid and comparably reliable to the satellite test, which requires a minimum of 24 h of incubation—representing one of the earliest documented applications of bacterially derived porphyrins as a diagnostic marker [34]. With the advent of advanced fluorescence imaging techniques, it became evident that the red autofluorescence observed in tissues largely originates from porphyrins, whose accumulation reflects either disturbances in heme biosynthesis or the metabolic activity of colonizing bacteria such as *Propionibacterium acnes* in the skin, *Pseudomonas aeruginosa* in wound infections, and various anaerobic species, including *Actinomyces odontolyticus* and *Bacteroides intermedius*, in the oral cavity. In experimental models of oral squamous cell carcinoma, Koenig and Schneckenburger were among the first to demonstrate that laser-induced tissue autofluorescence can serve as a practical, noninvasive diagnostic tool, with distinct spectral signatures reflecting the contributions of endogenous fluorophores, including bacterially and tissue-derived porphyrins [35]. On this basis, they further showed that red autofluorescence arising from porphyrin accumulation—particularly in microbially colonized or pathologically altered regions—constitutes a diagnostically relevant fluorescence marker across a range of tissue lesions.

Dietel and colleagues showed that many oral and gastrointestinal tract bacterial strains can efficiently convert exogenous ALA into fluorescent porphyrins, including protoporphyrin IX and water-soluble porphyrins formed mainly via aerobic photo-oxidation of porphyrinogen precursors. The fluorescent porphyrin profile varied between strains and even within a single species under different culture conditions [36].

DaCosta et al. first applied porphyrin fluorescence imaging in chronic wounds using the PRODIGI device (405 nm excitation, white-light and fluorescence modes), in which green tissue autofluorescence contrasts with red bacterial porphyrin emission and the cyan pyoverdine signal of *Pseudomonas aeruginosa*, allowing detection of clinically significant bacterial burden in the wound periphery and off-site areas usually missed during routine inspection [37].

The same principle—detection of bacterial porphyrin autofluorescence—proved effective for identifying dental calculus and was adapted for the VistaCam dental system. Teeth bearing supragingival and subgingival calculus were examined with this system, using 405 nm LED excitation to capture the porphyrin signal, yielding numerical scores that significantly distinguished calculus from both sound root surfaces and carious lesions, independent of saliva or blood contamination [38].

In an in vitro validation study, Jones et al. cultured 32 wound-pathogen bacterial species and four yeast species on ALA-containing Remel Porphyrin Test Agar and recorded fluorescence at 405 nm excitation (MolecuLight i:X). Red fluorescence was observed in 28 of 32 bacterial species (87.5%), in line with their porphyrin-producing capacity, was absent in the four non-porphyrin-producing species and was also detectable in polymicrobial biofilms [39].

In a retrospective clinical study, Price et al. analyzed foot ulcers imaged with the MolecuLight i:X under 405 nm excitation, recording bacterial porphyrin autofluorescence. Incorporating this method into routine practice was associated with a 33% reduction in antibiotic prescriptions and a 49% reduction in the prescription of antimicrobial dressings, together with a 23% relative increase in the proportion of wounds healed within 12 weeks (48% vs. 39%), thereby allowing for substantial cost savings [40].

Sun et al. cultured 15 bacterial and 2 fungal strains on Porphyrin Test Agar and blood agar, recording fluorescence with the wearable REVEAL FC system (405 nm excitation, 430 nm emission eyewear). Porphyrin-associated fluorescence was detected in most of the tested bacterial strains. These findings confirmed the sensitivity and convenience of wearable fluorescence technology and its potential for real-time, fluorescence-guided diagnosis of wound infection [21].

Ivanova et al. developed a mycobacterial detection method based on porphyrins, characterizing the spectral-fluorescent properties of 19 bacterial species, including 17 mycobacterial species (*M. tuberculosis*, *M. bovis*, *M. avium*, and others), across a range of concentrations and in mixed microbial associations using a spectrofluorometer at 633 nm excitation. Mycobacterial fluorescence remained weak even at high concentrations, which limited identification within polymicrobial communities. This limitation was overcome using a quaternary ammonium surfactant, benzyldimethyl [3-(myristoylamino) propyl] ammonium chloride (Miramistin), that releases intracellular porphyrins extracellularly, enhancing their detectability [41].

Endogenous bacterial porphyrins act as natural photosensitizers: upon absorption of visible-light photons, they are promoted to an excited singlet state and subsequently, through intersystem crossing, to a long-lived triplet state. By transferring energy to molecular oxygen, the excited porphyrins induce the generation of singlet oxygen and other reactive oxygen species, which cause irreversible oxidative damage to cellular structures and lead to bacterial cell death. Shleeva et al. likewise investigated the efficacy of photoinactivation in *Mycobacterium smegmatis*, whose dormant forms accumulate porphyrins similarly to *Mtb*. The authors induced the formation of dormant *M. smegmatis* forms through gradual acidification of the medium during the stationary phase (14 days). Cells were cultured either with or without the addition of 3 mM ALA. Photodynamic inactivation was performed by irradiation with a 532 nm laser, and viability was assessed by colony-forming unit counts. Irradiation of dormant cells produced a marked reduction in viability of approximately 2.5–4 log_10_ CFU, in contrast to actively growing cells, with the supplementation of exogenous ALA further enhancing photosensitivity [42].

In a subsequent study, Shleeva et al. demonstrated the efficacy of photoinactivation against *Mtb* itself. Dormant forms were obtained in modified Sauton medium under self-acidification over 30–50 days, with 3 mM ALA added on day 20. Photoinactivation was performed by irradiation with an LED SOLIS-4C light-emitting diode source fitted with a 565/24 nm filter (300 J/cm^2^ over 30 min). With the addition of exogenous ALA, irradiation produced greater than 99.99% killing of dormant *Mtb* cells in vitro [24].

### 3.2. ALA-Induced Porphyrin Fluorescence: Detection of Mtb and Discrimination from Contaminating Microorganisms

In this study, we demonstrated the feasibility of detecting ALA-induced porphyrins as indicators of *Mtb* growth and of discriminating *Mtb* from common contaminating microorganisms.

The addition of ALA to the culture medium led to a marked increase in the fluorescence intensity of the microorganisms studied over the course of cultivation. This effect can be attributed to the role of ALA as a precursor in the heme biosynthesis pathway: its exogenous supply promotes the accumulation of intermediate tetrapyrrole compounds—porphyrins, which exhibit characteristic red fluorescence.

Detection of *Mtb* growth relied on the 620 nm peak, whereas discrimination between *Mtb* and contaminating microorganisms was achieved through a combination of two parameters—the growth dynamics of the 620 nm peak intensity and the 580/620 nm intensity ratio. Emission at 685 nm was consistently recorded alongside the 580 and 620 nm maxima but was not included in the quantitative analysis, as under the conditions studied it proved to be the least informative of the three bands. Its increase during incubation lagged behind that of the 620 nm signal and therefore offered no advantage for the early detection of mycobacterial growth. At the same time, it did not separate *Mtb* from the contaminants tested as effectively as the growth dynamics of the 620 nm peak and the 580/620 nm ratio. The analysis was therefore restricted to these two parameters, which combined the earliest detectable response with the best discriminative capacity.

Both parameters proved informative at different stages of incubation and for different species, and it is precisely this complementarity that makes their combination reliable. *S. epidermidis* and *S. aureus* were distinguished from *Mtb* primarily by the early and pronounced increase in the 620 nm peak: by day 2, the 620 nm signal reached 132,933 and 110,625, respectively, whereas in *Mtb* it amounted to only 21,248, and by day 4 the signal of *S. aureus* was approximately 18-fold higher than that of *Mtb* (Table 3; *p* < 0.01). At later time points, the 580/620 nm ratio provided additional separation for these two species, exceeding 3 in *S. epidermidis* and approaching 0.94 in *S. aureus* by day 15, compared with 0.34–0.36 in *Mtb*.

For *C. albicans*, *P. aeruginosa*, *and K. pneumoniae*, the 620 nm peak either did not differ significantly from that of *Mtb* or was lower (Table 4, Table 5 and Table 6), and early differentiation was achieved mainly through the 580/620 nm ratio. *K. pneumoniae* and *P. aeruginosa* showed a clear, progressive increase in this ratio from day 3–4 onwards, reaching 1.66–2.85 by day 15. In *C. albicans*, the ratio observed on day 1 (0.92) corresponded to a 620 nm signal still close to the background level (2879), whereas a more pronounced and stable differentiation for this species emerged by day 4, when the ratio reached 0.41 at a substantially increased 620 nm signal (72,833). Thus, where the differences in one of the parameters were less pronounced, they were compensated for by the second, which together made it possible to reliably distinguish *Mtb* H37Rv from the five non-mycobacterial microorganisms studied under the experimental conditions used.

### 3.3. Practical Relevance

Conceptually, the proposed approach is related to the principles underlying automated culture systems, although a direct comparison with such systems was not among the objectives of the present study. One aspect of the findings may nevertheless be of practical interest. In automated systems—in particular the widely used BACTEC MGIT platform—contamination is essentially inferred from an implausibly early growth signal, since the instrument registers only the increase in fluorescence intensity. The approach considered here retains this criterion but adds an independent spectral parameter, the 580/620 nm ratio, thereby increasing the likelihood that contamination is recognized during incubation rather than retrospectively. Most of the contaminating microorganisms tested differed from *Mtb* before day 15 in at least one of the two parameters—fluorescence intensity or the 580/620 nm ratio—that is, within a period in which mycobacterial growth is often not yet detected. This is of practical relevance, since contamination developing from a low initial inoculum may not produce an early growth signal and would otherwise be recognized only after the tube has been removed from the instrument.

### 3.4. Methodological Considerations

Several methodological aspects of the present study warrant consideration when interpreting these findings. In accordance with standard laboratory procedures, inocula were standardized to 0.5 McFarland turbidity for all strains. It should be noted, however, that this turbidity does not always correspond to strictly equivalent CFU/mL across species, as the relationship between optical density and the number of viable cells depends on cell size, morphology, and clumping tendency—this is particularly characteristic of mycobacteria compared with other bacteria. Thus, it cannot be excluded that differences in the initial cell numbers may have affected the results obtained.

It should also be noted that the selective solid media (mannitol salt agar, MacConkey agar, and Sabouraud agar) were used only to obtain pure cultures for the preparation of the inocula, whereas the incubation in the experiment itself was carried out under identical conditions for all microorganisms, including *Mtb*—in Middlebrook 7H9 medium supplemented with OADC enrichment, at the same ALA concentration, and under the same incubation regimen. All spectral measurements were therefore performed in the same medium so that the observed differences in the fluorescence patterns cannot be attributed to differences in medium composition. Nevertheless, differences in the initial metabolic state of the cells at the moment of inoculation, resulting from the preceding cultivation on different selective media, cannot be completely excluded and may have influenced the early stages of ALA conversion.

### 3.5. Directions for Further Research

In the present study, the reference strain *Mtb* H37Rv was used, as it provides reproducible and readily interpretable baseline data, consistent with the aim of this stage—establishing the principal feasibility of the approach on a well-characterized model system. Building on these results, a natural next step will be to expand the panel of tested strains to include clinical isolates, drug-resistant strains, and non-tuberculous mycobacteria, which will allow the applicability of the approach to be assessed across a broader range of clinically relevant scenarios. Although species-level identification was beyond the scope of the present work, the potential of the approach makes it reasonable to explore the detection of NTM and to search for spectral differences between *Mtb*, NTM, and opportunistic microorganisms that could support species-level differentiation.

A separate direction for further work is a systematic evaluation of the concentration–response relationship on an adequately sized sample, covering fluorescence intensity, the 580/620 nm ratio, growth kinetics, and reproducibility. Such an evaluation will make it possible to define the concentration range providing the maximum useful signal, to clarify whether the 580/620 nm ratio itself depends on the concentration of the porphyrin precursor, and to establish the minimal effective concentration, thereby improving the cost-efficiency of the approach.

Finally, a more detailed mechanistic characterization—including direct measurements of ALA uptake, porphyrin concentrations, heme-pathway intermediates, and enzyme activities—would help to identify the rate-limiting steps of ALA-induced porphyrin biosynthesis and thus provide a rational basis for further refinement of the approach. The results presented here are intended to provide an experimental basis for further investigation and optimization of this approach.

## 4. Materials and Methods

### 4.1. Microorganisms and Culture Conditions

The laboratory strain *Mtb* H37Rv, obtained from the collection of the Microbiology Laboratory of the National Medical Research Center for Phthisiopulmonology and Infectious Diseases, was used in this study. The strain was cultured in the automated BACTEC MGIT 960 system in liquid Middlebrook 7H9 (Becton Dickinson Diagnostic Systems, Sparks, MD, USA) medium supplemented with OADC enrichment until a positive growth signal was obtained. After growth positivity was detected, an *Mtb* H37Rv suspension was prepared and adjusted to a turbidity of 0.5 McFarland.

**Figure 8:**
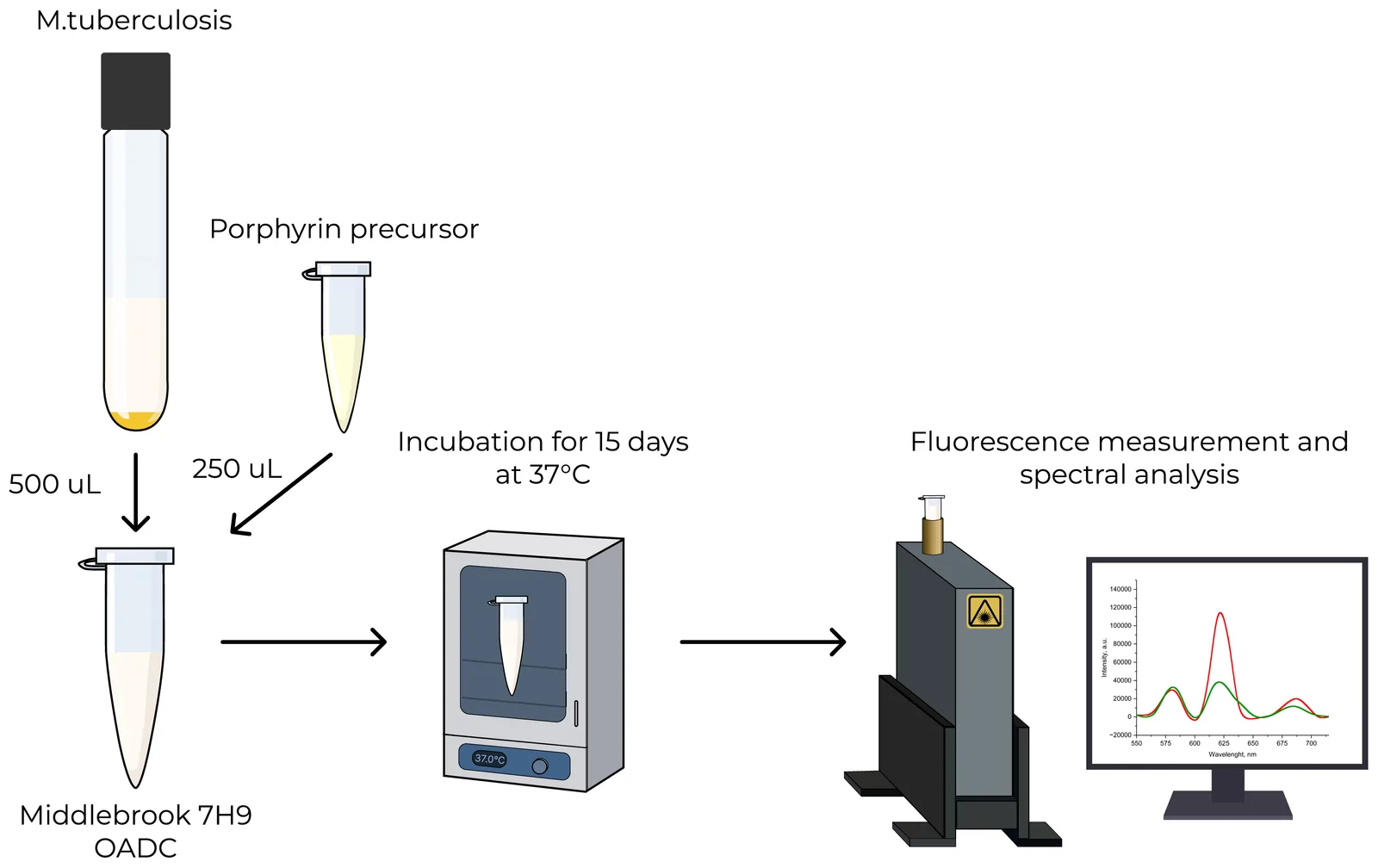
Scheme of experiment. A 500 µL suspension of *M. tuberculosis* H37Rv in Middlebrook 7H9 medium supplemented with OADC was mixed with 250 µL of the fluorescence inducer. Samples were incubated at 37 °C for 15 days. Fluorescence measurements and spectral analysis were subsequently performed. The representative spectra shown in the inset illustrate the fluorescence profiles of *M. tuberculosis* H37Rv (red line) and *P. aeruginosa* (green line). OADC, oleic acid, albumin, dextrose, and catalase.

Cultures of *S. epidermidis* and *S. aureus* were pre-cultured on mannitol salt agar, *K. pneumoniae* and *P. aeruginosa* on MacConkey agar, and *Candida albicans* on Sabouraud agar. Incubation was performed at 37 °C for 24 h. The resulting isolated colonies were used to prepare microbial suspensions with a turbidity of 0.5 McFarland in Middlebrook 7H9 medium supplemented with OADC (Figure 8).

### 4.2. Preparation of ALA-Containing Samples

ALA dissolved in Middlebrook 7H9 medium supplemented with OADC was used as a precursor for porphyrin synthesis. The working ALA solution was added to culture tubes in a volume of 250 µL at a final concentration of 4 mM.

Negative controls (Middlebrook 7H9 medium with OADC and ALA, without mycobacterial suspension) were included to exclude false-positive results.

### 4.3. Experimental Design

Briefly, 500 µL suspension of *Mtb* H37Rv in Middlebrook 7H9 medium supplemented with OADC was inoculated with 250 µL of ALA solution, yielding a final volume of 750 µL per tube. The same sample variants were prepared for *S. epidermidis*, *S. aureus*, *K. pneumoniae*, *P. aeruginosa*, and *C. albicans*. Middlebrook 7H9 medium supplemented with OADC (500 µL) and ALA (250 µL), with a final volume of 750 µL, served as a negative control. After preparation, all tubes were incubated at 37 °C for 15 days. All the above-mentioned bacterial strains were tested in 10 independent experiments each.

### 4.4. Fluorescence Measurements

The fluorescence signal was recorded once daily at the same time for all samples. Fluorescence measurements were performed using an Enspectr L405 portable luminescencespectrometer (Enhanced Spectrometry, Inc., Austin, TX, USA) at an excitation wavelength of 405 nm and an output power of 8 mW. The signal accumulation time was 2 s, with measurements taken through the bottom of the tube.

### 4.5. Statistical Analysis

Continuous variables (peak intensity at 620 nm and the 580/620 nm ratio) are presented as medians, since the distributions of the measured parameters deviated from normality. To compare fluorescence intensity between *Mtb* and other species cultures across the repeated measurement time points, a repeated-measures analysis of variance (ANOVA), with species and time as factors, was applied in jamovi (v2.7.38.0; The jamovi Project, Sydney, Australia). Statistical significance was set at *p* < 0.05.

## 5. Conclusions

The proposed approach demonstrated, as a proof of principle, the feasibility of mycobacterial detection via porphyrin-based fluorescence, leveraging the heme biosynthesis pathway as the mechanistic basis for the development of a novel detection strategy for *Mtb*. Exogenous supplementation with the porphyrin precursor 5-aminolevulinic acid hydrochloride yielded a pronounced enhancement of the fluorescence signal, substantially increasing the sensitivity of the method. Furthermore, the spectral characteristics of mycobacteria were compared against a panel of opportunistic microorganisms that may act as potential contaminants, revealing distinct spectral profiles that differentiate *Mtb* from these microorganisms and offering additional potential for the identification of sample contamination. Taken together, these findings establish an initial proof-of-principle for a novel method of *Mtb* detection in tuberculosis, integrating heme biosynthesis pathway induction with subsequent spectrofluorimetric analysis. Further validation will necessitate investigation of clinical specimens, as well as evaluation of a broader range of contaminating microorganisms, to establish the feasibility of discriminating *Mtb* from other microorganisms.

## Figures and Tables

**Table 1:** Fluorescence signal measurements during *Mtb* culture.

Day	Peak at 580 nm, Median	Peak at 620 nm, Median	580/620 nm Ratio, Median
1	0	14,865	0.00
2	0	21,248	0.00
3	0	30,324	0.00
4	6330	38,762	0.17
5	10,718	58,736	0.20
6	15,283	70,675	0.21
7	19,456	86,467	0.22
8	23,879	95,364	0.25
9	28,620	112,515	0.25
10	43,703	175,231	0.26
11	67,527	216,930	0.3
12	84,463	258,428	0.32
13	106,054	299,987	0.34
14	137,334	382,933	0.37
15	147,767	419,993	0.36

**Table 2:** Median fluorescence signal intensities of *S. epidermidis* at 580 and 620 nm.

Day	Peak at 580 nm, Median	Peak at 620 nm, Median	580/620 nm Ratio, Median
1	2282	20,502	0.11
2	9283	132,933	0.07
3	41,610	406,643	0.10
4	81,412	357,676	0.24
5	135,543	361,036	0.38
6	197,307	389,696	0.51
7	252,593	396,001	0.67
8	293,080	489,025	0.58
9	331,476	459,959	0.75
10	361,310	386,315	0.97
11	359,545	338,181	1.06
12	357,780	290,047	1.35
13	361,938	263,301	1.52
14	396,911	248,865	1.64
15	359,889	215,207	1.74

**Table 3:** Median fluorescence signal intensities of *S. aureus* at 580 and 620 nm.

Day	Peak at 580 nm, Median	Peak at 620 nm, Median	580/620 nm Ratio, Median
1	2005	20,396	0.11
2	6953	110,625	0.06
3	35,107	373,228	0.09
4	75,892	518,881	0.14
5	102,360	540,188	0.19
6	110,917	599,584	0.19
7	172,089	555,489	0.31
8	204,026	437,480	0.39
9	222,502	526,885	0.43
10	232,755	483,345	0.52
11	240,663	434,894	0.55
12	248,570	386,443	0.58
13	272,914	377,127	0.69
14	295,027	341,851	0.84
15	304,186	323,395	0.94

**Table 4:** Median fluorescence signal intensities of *K. pneumoniae* at 580 and 620 nm.

Day	Peak at 580 nm, Median	Peak at 620 nm, Median	580/620 nm Ratio, Median
1	1479	9401	0.25
2	2036	16,042	0.21
3	3323	24,460	0.37
4	9522	29,986	0.67
5	19,816	35,436	0.81
6	29,643	40,073	1.05
7	42,218	46,735	1.27
8	63,038	46,124	1.54
9	75,979	46,708	1.71
10	80,769	41,886	1.82
11	80,054	43,140	1.94
12	83,522	44,394	1.89
13	86,990	46,680	1.81
14	93,298	62,485	1.58
15	117,813	70,627	1.66

**Table 5:** Median fluorescence signal intensities of *C. albicans* at 580 and 620 nm.

Day	Peak at 580 nm, Median	Peak at 620 nm, Median	580/620 nm Ratio, Median
1	0	2879	0.92
2	2967	8513	1.25
3	7820	36,406	0.33
4	14,004	72,833	0.41
5	25,311	99,022	0.41
6	45,202	110,421	0.54
7	62,011	109,174	0.70
8	81,547	107,860	0.71
9	91,839	140,425	0.82
10	131,701	139,153	0.94
11	158,158	158,588	1.15
12	182,424.5	178,022	1.10
13	206,691	205,635	1.14
14	239,322	253,910	1.01
15	269,164	257,635	1.16

**Table 6:** Median fluorescence signal intensities of *P. aeruginosa* at 580 and 620 nm.

Day	Peak at 580 nm, Median	Peak at 620 nm, Median	580/620 nm Ratio, Median
1	1961	9793	0.21
2	2489	11,859	0.20
3	3740	20,961	0.19
4	5640	19,814	0.30
5	6322	25,232	0.24
6	11,420	33,611	0.32
7	24,314	36,035	0.70
8	30,530	33,300	1.05
9	40,910	32,669	1.31
10	51,492	29,045	1.68
11	59,382	29,806	1.99
12	67,272	30,566	2.23
13	71,797	29,166	2.51
14	77,396	32,582	2.79
15	78,145	27,035	2.85

## Data Availability

The original contributions presented in this study are included in the article. Further inquiries can be directed to the corresponding author.

## Abbreviations

The following abbreviations are used in this manuscript:

ALA	5-aminolevulinic acid
CPD	Coproporphyrin-dependent pathway
CFU	Colony-forming unit
*Mtb*	*Mycobacterium tuberculosis*
NTM	Nontuberculous mycobacteria
TB	Tuberculosis
WHO	World Health Organization
PCR	Polymerase chain reaction
OADC	Oleic acid, albumin, dextrose, catalase
